# 5-HT_3_ receptor antagonists decrease the prevalence of postoperative delirium in older patients undergoing orthopedic lower limb surgery

**DOI:** 10.1186/s13741-021-00222-3

**Published:** 2021-12-08

**Authors:** Hyun-Jung Shin, Jiwon Yoon, Hyo-Seok Na

**Affiliations:** Department of Anesthesiology and Pain Medicine, Seoul National University Bundang Hospital, 82, Gumi-ro 173 Beon-gil, Bundang, Seongnam, 13620 South Korea

**Keywords:** 5-HT_3_ receptor antagonist, Postoperative delirium, Older patient

## Abstract

**Background:**

Delirium is an important postoperative complication. Recent research suggested that 5-hydroxytryptamine 3 (5-HT_3_) receptor antagonists may have clinical effect in the treatment and prevention of delirium. We investigated the association between 5-HT_3_ receptor antagonists and the occurrence of postoperative delirium (POD).

**Methods:**

Retrospectively, the electronic medical records were reviewed in patients aged ≥ 65 years who underwent orthopedic lower limb surgery under regional anesthesia (spinal or combined spinal-epidural anesthesia) and administered intravenous 0.075 mg palonosetron or 0.3 mg ramosetron prior to the end of surgery between July 2012 and September 2015. POD incidence and anesthesia-, surgery-, and patient-related factors were evaluated. To investigate the association between 5-HT_3_ receptor antagonists and the occurrence of POD, multivariable logistic regression analysis was performed.

**Results:**

Of the 855 patients included, 710 (83%) were administered 5-HT_3_ receptor antagonists. POD was confirmed in 46 (5.4%) patients. 5-HT_3_ receptor antagonists reduced the POD incidence by 63% (odds ratio [OR] 0.37; 95% confidence interval [CI], 0.15–0.94; *P* = 0.04). Moreover, the POD incidence decreased by 72% (OR 0.28, 95% CI 0.10–0.77, *P* = 0.01) when palonosetron was administered. Other identified risk factors for POD were emergency surgery, older age, hip surgery, lower body mass index, and intraoperative propofol sedation.

**Conclusion:**

5-HT_3_ receptor antagonists may be related with a significantly reduced risk for POD in older patients undergoing orthopedic lower limb surgery. Notably, palonosetron was more effective for POD prevention.

## Introduction

In older patients, postoperative delirium (POD) is an important complication, with a reported incidence of 5–61% following orthopedic surgery (Morrison et al. 1998; Rade et al. 2011; Robertson and Robertson 2006). It has been related with higher mortality and morbidity rates, prolonged hospital stays, and delayed functional recovery (Liang et al. 2014; Rade et al. 2011; Robertson and Robertson 2006). Consequently, it has received increasing attention over the last two decades as an important challenge for clinicians participated in the perioperative care of older patients. Due to its multifactorial etiology, POD is difficult to prevent or treat (Moyce et al. 2014). Nonetheless, related study has continued to identify the related risk factors with the purpose to develop an optimal strategy for its prevention and treatment (Bitsch et al. 2004; Shin et al. 2017).

To minimize the incidence of postoperative nausea and/or vomiting (PONV), antagonists of the 5-hydroxytryptamine receptor subtype 3 (5-HT_3_) have been administered (Smith et al. 2012). Recently, 5-HT_3_ receptor antagonists have received attention as pharmacological options for the treatment and prevention of delirium (Lirk and Hollmann 2014; Papadopoulos et al. 2014; Tagarakis et al. 2012). Namely, 5-HT not only regulates body temperature, sleep, and pain but also modulates cognition and emotional behavior (Svob Strac et al. 2016), and numerous clinical and preclinical researches have found that antagonists of various 5-HT receptor subtypes significantly improve cognition (Bayndr et al. 2000; Qiu et al. 2016). Although some clinical researches have reported beneficial effects of 5-HT_3_ receptor antagonist use on the occurrence of POD in surgical patients (Bayndr et al. 2000; Papadopoulos et al. 2014), data on their association are still scarce. In addition, most previous clinical researches of 5-HT_3_ receptor antagonists have focused on POD after cardiac surgery (Bayndr et al. 2000; Tagarakis et al. 2012), with few studies investigating their effects after orthopedic surgery (Papadopoulos et al. 2014).

Therefore, the purpose of this study was to determine whether 5-HT_3_ receptor antagonist administration affected the risk of POD after orthopedic lower limb surgery with regional anesthesia.

## Methods

### Study setting and patients

This historical cohort study was approved by the Institutional Review Board of Seoul National University Bundang Hospital (B-1903/531-107, February 12, 2020) and performed according to institutional guidelines. An informed consent requirement was waived.

The electronic medical records were reviewed retrospectively in patients aged ≥ 65 years who underwent orthopedic lower limb surgery under regional anesthesia (spinal or combined spinal-epidural anesthesia) from July 2012 to September 2015. The included surgeries (International classification of Disease-9-Clinical Modification Procedure Codes) were as follows: (1) open reduction and internal fixation of fracture (femur, 79.35; tibia and fibula, 79.36), (2) closed reduction and internal fixation of fracture (femur, 79.15; tibia and fibula, 79.16), (3) joint replacement with an artificial or mechanical device or prosthesis (hip, v43.64; knee, v43.65; ankle, v43.66). Patients with preoperatively impaired cognitive function, those with incomplete medical records, and patients with intraoperative anesthesia conversion to general anesthesia were excluded.

### Perioperative 5-HT_3_ receptor antagonists use

Intravenous 0.075 mg palonosetron or 0.3 mg ramosetron were administered prior to the end of the surgery for prevention of PONV. As a rescue antiemetic agent, metoclopramide was used after surgery.

### Assessment of postoperative abnormal behavior

Orthopedic surgeons sought consultation from psychiatrists if the patients showed symptoms suggestive of POD, like anxiety, agitation, irritability, fear, apathy, depression, incontinence, suspicion, euphoria, and hallucinations. Psychiatrists immediately evaluated the patients using the Confusion Assessment Method for the intensive care unit (Inouye et al. 1990) and prescribed pertinent therapy in cases where POD was diagnosed.

### Data collection

The following three sets of variable data were collected and categorized into the following: (1) preoperative factors, such as preoperative laboratory findings, age, gender, height, weight, body mass index, American Society of Anesthesiologists physical status (ASA-PS) class, and Charlson comorbidity index; (2) intraoperative factors, such as surgery and anesthesia duration, type of surgery (elective or emergency), sedatives used for intraoperative sedation, use of midazolam for premedication, use of vasopressors, and red blood cell (RBC) transfusion; and (3) postoperative factors, such as postoperative laboratory findings, occurrence of POD, intensive care unit (ICU) admittance, postoperative hospital stay, and RBC transfusion. Pre- and postoperative laboratory tests included electrolytes, creatinine, albumin, and hematocrit levels.

### Endpoints

The primary endpoint was the association of 5-HT_3_ receptor antagonist use with the incidence of POD in older patients undergoing orthopedic lower limb surgery under regional anesthesia. Additionally, we examined other risk factors related to POD.

### Statistical analysis

The patients’ baseline characteristics were presented as means with standard deviations or numbers with percentages. Multivariable logistic regression analysis was used to evaluate the association between 5-HT_3_ receptor antagonist use and the incidence of POD. We constructed the initial multivariable logistic model using all variables (gender, age, weight, height, body mass index, Charlson comorbidity index, ASA-PS class, hypertension, diabetes mellitus, history of stroke, chronic kidney disease, intraoperative sedatives, midazolam premedication, operation time, RBC transfusion, urgency of surgery, vasopressor use, laboratory findings, ICU admittance, and 5-HT_3_ receptor antagonist use) and then applied the backward variable-elimination method to fit the final multivariable logistic model to identify the significant factors related to POD. To avoid multicollinearity, the use and type of 5-HT_3_ receptor antagonists were included in each multivariable logistic regression model.

All statistical analyses were conducted using IBM® SPSS® Statistics version 22.0 (IBM Corporation, Armonk, NY, USA) or Stata® SE version 14 (StataCorp LLC, College Station, TX, USA). *P* values less than 0.05 were considered as statistically significant.

## Results

### Patients’ characteristics

**Fig. 1:**
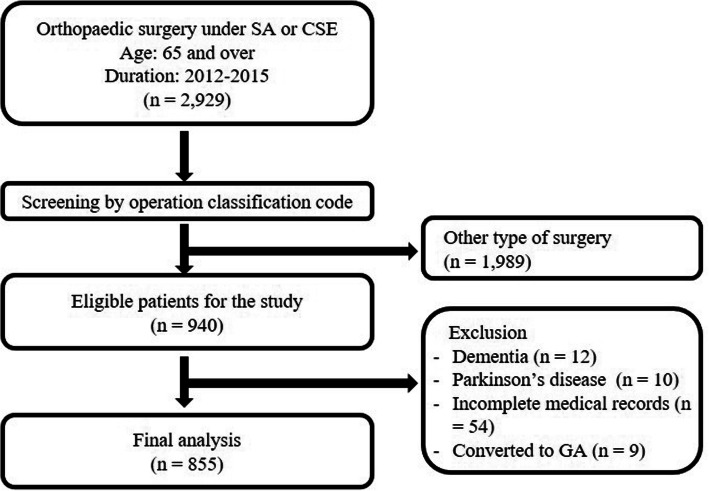
Flow chart. SA, spinal anesthesia; CSE, combined spinal-epidural anesthesia; GA, general anesthesia

Of the 2929 patients evaluated for eligibility, 855 met the study criteria (Fig. 1). The characteristics of the patients are described in Table 1.

**Table 1:** Baseline characteristics of the total study population

	Patients		
Variable	Total (*n* = 855)	No delirium (*n* = 809)	Delirium (*n* = 46)
Age (year)	73 (5)	73 (5)	78 (6)
Gender (female)	739 (86.4%)	707 (87.4%)	32 (69.6%)
Height (cm)	154 (7)	154 (7)	156 (9)
Weight (kg)	63 (9)	63 (9)	59 (9)
Body mass index (kg/m^2^)	27 (4)	27 (4)	25 (4)
ASA class			
1	115 (13.5%)	112 (13.8%)	3 (6.5%)
2	699 (81.8%)	662 (81.8%)	37 (80.4%)
3	41 (4.8%)	35 (4.3%)	6 (13%)
Charlson comorbidity index	3.8 (0.9)	3.8 (0.9)	4.4 (1.0)
Hypertension	600 (70.2%)	566 (70.0%)	34 (73.9%)
Diabetes mellitus	228 (26.7%)	210 (26.0%)	18 (39.1%)
History of stroke	37 (4.3%)	32 (4.0%)	5 (10.9%)
Chronic kidney disease	31 (3.6%)	25 (3.1%)	6 (13.0%)
Intraoperative sedative			
Propofol	592 (69%)	552 (68%)	40 (87%)
Dexmedetomidine	263 (31%)	257 (32%)	6 (13%)
Midazolam premedication	836 (97.8%)	792 (97.9%)	44 (95.7%)
Operation time (min)	114 (46)	115 (46)	99 (47)
Anesthesia time (min)	162 (54)	163 (54)	148 (52)
RBCs (units, during surgery)	0.1 (0.5)	0.1 (0.5)	0.1 (0.3)
RBCs (units, after surgery)	0.2 (0.6)	0.2 (0.6)	0.5 (0.9)
Emergency operation	13 (1.5%)	9 (1.1%)	4 (8.7%)
Phenylephrine (μg)	21 (54)	20 (53)	39 (64)
Ephedrine (mg)	5.2 (7.0)	5.3 (7.0)	4.4 (5.5)
Laboratory findings-pre			
Hematocrit (%)	36.6 (5.1)	36.7 (5.1)	35.6 (5.7)
Sodium (mmol/L)	141 (3)	141 (3)	140 (3)
Potassium (mmol/L)	4.3 (0.4)	4.3 (0.4)	4.4 (0.6)
Creatinine (mg/dL)	0.8 (0.5)	0.8 (0.4)	1.1 (1.1)
Albumin (g/dL)	4.0 (0.5)	4.0 (0.5)	3.9 (0.6)
Laboratory findings-post			
Hematocrit (%)	29.9 (4.1)	30.0 (4.1)	29.2 (3.5)
Sodium (mmol/L)	138 (3)	138 (3)	136 (3)
Potassium (mmol/L)	4.2 (0.4)	4.2 (0.4)	4.3 (0.5)
Creatinine (mg/dL)	0.7 (0.4)	0.7 (0.4)	1.1 (1.0)
Albumin (g/dL)	3.4 (0.3)	3.4 (0.3)	3.2 (0.4)
Postoperative ICU admittance	15 (1.8%)	10 (1.2%)	5 (10.9%)
Postoperative admission period (days)	14 (11)	14 (9)	18 (23)
5-HT_3_ receptor antagonist			
None	145 (17%)	137 (17%)	8 (18%)
Ramosetron	365 (43%)	346 (43%)	19 (41%)
Palonosetron	345 (40%)	326 (40%)	19 (41%)
Delirium	46 (5.4%)	46 (100%)	N/A
Delirium onset (days)	1.7 (1.5)	1.7 (1.5)	N/A

Data are expressed as the mean (SD) or the number of the patients (proportion)

*BMI* body mass index, *ASA* American Society of Anesthesiologist, *RBC* red blood cell, *ICU* intensive care unit, *5-HT* 5-hydroxytryptamine, *N/A* not applicable

### Association between 5-HT_3_ receptor antagonist use and POD occurrence

The total incidence of POD was 5.4% (46 patients) and the ICD-9 code was 293.0 (delirium due to conditions classified elsewhere) in all diagnosed patients. POD was diagnosed in 19 (13.1%) patients who did not receive 5-HT_3_ receptor antagonists, 19 (5.2%) patients who received ramosetron, and 8 (2.3%) patients who received palonosetron.

The ICD-9 codes of surgery in 46 patients diagnosed POD were as follows *n* (%): v43.64 = 20 (43.5%); v43.65 = 17 (37.0%); 79.15 = 4 (8.7%); 79.35 = 3 (6.5%); and 79.36 = 2 (4.3%).

The final results of the multivariable logistic regression analysis are shown in Table 2. Patients who received 5-HT_3_ receptor antagonists had a 63% lower incidence of POD compared with those who did not receive these drugs (odds ratio [OR], 0.37; confidence interval [CI], 0.15–0.94; *P* = 0.04). Notably, palonosetron use reduced the incidence of POD by 72% (OR, 0.28; 95% CI, 0.10–0.77; *P* = 0.01), while the use of ramosetron did not show significant effects on the incidence of POD (*P* = 0.16).

**Table 2:** Multivariable logistic regression analysis for postoperative delirium

Variable	Multivariate logistic model		
	OR	95% CI	*P* value
Age	1.11	1.04–1.79	0.002
BMI	0.89	0.79–0.98	0.016
Charlson comorbidity index	1.95	1.30–2.93	0.001
Urgency of surgery			
Elective	1		
Emergency	7.84	1.55–39.64	0.013
Type of surgery			
Non-hip	1		
Hip	6.91	3.25–14.66	< 0.001
Sedative			
Propofol	1		
Dexmedetomidine	0.20	0.08–0.58	0.002
5-HT_3_ receptor antagonists			
No	1		
Yes	0.37	0.15–0.94	0.036
5-HT_3_ receptor antagonists			(0.047)
None	1		
Ramosetron	0.48	0.17–1.33	0.157
Palonosetron	0.28	0.10–0.77	0.014

For final multivariable model, backward variable-selection method was performed (removed variables: gender, ASA classification, hypertension, diabetes mellitus, history of stroke, chronic kidney disease, midazolam premedication, operation time, RBC transfused, phenylephrine, ephedrine, laboratory findings, and ICU admittance)

*CI* confidence interval, *BMI* body mass index, *5-HT* 5-hydroxytryptamine, *ASA* American Society of Anesthesiologists, *RBC* red blood cell, *ICU* intensive care unit

### Other factors associated with POD occurrence

Other factors associated with the occurrence of POD included age (OR 1.11, 95% CI 1.04–1.79, *P* = 0.002), body mass index (OR 0.89, 95% CI 0.79–0.98, *P* = 0.02), Charlson comorbidity index (OR 1.95, 95% CI 1.30–2.93, *P* = 0.001), emergency surgery (OR 7.84, 95% CI 1.55–39.63, *P* = 0.01), hip surgery (OR 6.91, CI 3.25–14.66, *P* < 0.001), and dexmedetomidine use (OR 0.21, 95% CI 0.07–0.58, *P* = 0.002).

## Discussion

In the present study, we found that older patients who received 5-HT_3_ receptor antagonists had a lower incidence of POD than those who did not receive these drugs. To the best of our knowledge, this is the first study to verify the association between 5-HT_3_ receptor antagonist use and the occurrence of POD in older patients who underwent orthopedic lower limb surgery. These findings are relevant because they provide important information regarding the delirium-sparing effect of 5-HT_3_ receptor antagonists at a time when the debates continue regarding the pharmacological prevention of POD (Gosch and Nicholas 2014; Schwartz et al. 2016; Serafim et al. 2015).

Acetylcholine, dopamine, noradrenaline, and 5-HT are the neurotransmitters that have most often been involved in the genesis of delirium (Trzepacz 2000). Among them, 5-HT regulates higher brain functions, such as emotional behavior and cognition (Ciranna 2006). Nonetheless, although 5-HT_3_ receptors are distributed in brain areas that play a potential role in cognitive function (Hoyer et al. 2002), few clinical studies have investigated their involvement in cognition.

The cognitive-enhancing effects of ondansetron were tested in older rhesus monkeys (Arnsten et al. 1997). Another 5-HT_3_ receptor antagonist, tropisetron, alleviated deficits of spatial memory in a rat model of Alzheimer’s disease (Rahimian et al. 2013; Spilman et al. 2014). Clinically, the effect of 5-HT_3_ receptor antagonists on cognition has mostly been investigated in patients with schizophrenia (Ellenbroek and Prinssen 2015). In the treatment of schizophrenia, ondansetron has shown some effect, especially for cognitive impairments (Akhondzadeh et al. 2009; Zhang et al. 2006). Levkovitz et al. (2005) observed improvements of memory and visuo-spatial learning after ondansetron therapy, whereas verbal memory was improved following treatment with tropisetron (Zhang et al. 2012). These findings suggest that there may be a therapeutic potential for managing cognitive deficits with 5-HT_3_ receptor antagonists.

Although clinical researches are insufficient, 5-HT_3_ receptor antagonists have been known to provide a protective effect against cognitive dysfunction after surgery (Bayndr et al. 2000; Papadopoulos et al. 2014; Tagarakis et al. 2012). Papadopoulos et al. (Papadopoulos et al. 2014) investigated whether postoperative administration of ondansetron had a preventive effect on POD after orthopedic surgery. In their study, the ondansetron group showed a lower incidence and duration of POD compared with the placebo group. Tagarakis et al. (Tagarakis et al. 2012) compared the efficacy of haloperidol and ondansetron in treating POD after cardiac surgery and reported that both drugs had similar delirium-controlling effects. In another study performed in cardiac surgery patients, ondansetron showed a delirium-treating effect without serious side effects (Bayndr et al. 2000). Our results are in line with the findings of these clinical trials, showing a 63% reduction in the incidence of POD in patients administered 5-HT_3_ receptor antagonists.

We also identified the effects of different 5-HT_3_ receptor antagonists on POD prevention. Palonosetron showed more prominent effect in decreasing the occurrence of POD than ramosetron. Ramosetron has more potent and longer duration via greater binding affinity and slower dissociation on 5-HT_3_ receptor (Rabasseda 2002). Palonosetron belongs to the second-generation 5-HT_3_ receptor antagonists, which have been shown to have a greater receptor-binding affinity than that of the first-generation drugs (Smith et al. 2012). In addition, it has a longer plasma half-life than that of ramosetron (40 h vs. 9 h) (Gan 2005; Stoltz et al. 2004). More importantly, binding of palonosetron to the receptors triggers 5-HT_3_ receptor internalization, suggesting the ability of prolonged inhibition of the receptor function (Rojas et al. 2010). These features of palonosetron may explain its better efficacy in reducing the risk for POD compared to that of ramosetron. However, to confirm this difference, further studies are needed.

Previous researches have found multiple risk factors for POD, including advanced age, type of surgery, impaired cognitive status, high perioperative transfusion requirement, preoperative albumin level, and use of benzodiazepines (Bilotta et al. 2013; Shin et al. 2017; Steiner 2011). Our results showed similar related factors, including older age, higher Charlson comorbidity index, emergency surgery, and hip surgery. However, these are non-modifiable factors. Therefore, to prevent POD in high-risk patients, aggressive control of modifiable factors is important.

This study had several limitations resulting from its retrospective design. First, the number of factors influencing POD could not be controlled. For example, 97.8% of patients received intravenous midazolam preoperatively. Because benzodiazepine is regarded as a predisposing factor to POD (Steiner 2011), we cannot disregard the effect of midazolam on the overall incidence of POD in the present study. However, benzodiazepine increased the risk of delirium when it was given continuously rather than intermittently (Zaal et al. 2015). Therefore, we assume that just a single injection of midazolam had rarely influenced the occurrence of POD. Second, selection bias may have been present. However, to overcome this limitation, all medical data were extracted by a medical record expert who was blinded to the purpose of this study. Third, although patients with preoperatively impaired cognitive function were excluded, most of the patients did not assess their cognitive status using mental state examination tools, which could influence on the results. Fourth, because of the nature of retrospective design, not all patients evaluated their cognitive status every day after surgery. Thus, the incidence of POD might be underdiagnosed, especially hypoactive delirium form. Fifth, not all contributing factors which have been known to influence on POD, such as postoperative hypoxia, could include in the present analysis because the monitoring of oxygen saturation is not a routine practice at general ward. Sixth, patients who received general anesthesia were excluded. There are ongoing debates regarding the effect of anesthesia technique (regional vs. general) on POD (Patel et al. 2018). Because the purpose of the present study was to evaluate the effect of the 5-HT_3_ receptor antagonists on POD, we included regional anesthesia only in this study to minimize the confounding effect of general anesthesia. Finally, due to the study design (a single-center study), our results cannot be generalized.

In conclusion, our findings suggest that the use of 5-HT_3_ receptor antagonists is associated with a significantly lower risk for POD in older patients undergoing orthopedic lower limb surgery via spinal or combined spinal-epidural anesthesia. Furthermore, palonosetron demonstrated a significant efficacy in reducing POD incidence and may be considered for POD prevention in these patients. Nonetheless, further prospective studies are required to confirm the actual decrease in the occurrence of POD according to the use of different 5-HT_3_ receptor antagonists in both regional and general anesthesia.

## Data Availability

The datasets generated and/or analyzed during the current study are not publicly available due to the regulation of Institutional Review Board but are available from the corresponding author after getting permission from IRB for sharing the dataset on reasonable request.

## Abbreviations

ASA-PS: American Society of Anesthesiologists physical status
ICU: Intensive care unit
5-HT_3_: 5-Hydroxytryptamine 3
POD: Postoperative delirium
PONV: Postoperative nausea and/or vomiting
RASS: Richmond Agitation and Sedation Scale
RBC: Red blood cell
